# Professional burnout and patient safety culture in Primary Health Care

**DOI:** 10.1590/0034-7167-2022-0311

**Published:** 2023-08-07

**Authors:** Vitória Talya dos Santos Sousa, Hirlana Girão Dias, Fernanda Pereira de Sousa, Roberta Meneses Oliveira, Edmara Chaves Costa, Patrícia Freire de Vasconcelos

**Affiliations:** I Universidade da Integração Internacional da Lusofonia Afro-Brazileira. Redenção, Ceará, Brazil; II Universidade Federal do Ceará. Fortaleza, Ceará, Brazil

**Keywords:** Occupational Burnout, Patient Safety, Primary Health Care, Health Personnel, Organizational Culture., Agotamiento Profesional, Seguridad del Paciente, Atención Primaria de Salud, Personal de Salud, Cultura Organizacional., Esgotamento Profissional, Segurança do Paciente, Atenção Primária à, Saúde, Pessoal de Saúde, Cultura Organizacional.

## Abstract

**Objectives:**

to analyze the association between the risk of occupational exhaustion (burnout) and safety culture in Primary Health Care.

**Methods:**

ross-sectional study conducted in 18 Primary Health Care Units in the Northeast of Brazil. Three questionnaires were used: sociodemographic, Maslach Burnout Inventory, and the Medical Office Survey on Patient Safety Culture. The study was approved by the Research Ethics Committee.

**Results:**

seventy-eight healthcare workers participated, of which 64.1% presented a reduced risk of burnout; and 11.5%, a high risk (p=0.000). The following were identified as weakened dimensions of safety culture: Work pressure and pace; Owner, managing partners, leadership support; Overall ratings on quality; and Overall rating on patient safety.

**Conclusions:**

an association was found between low risk of developing burnout syndrome and positive evaluation of safety culture.

## RESUMO

**Objetivos:**

analisar a associação entre risco de esgotamento profissional (*burnout*) e cultura de segurança na Atenção Primária à Saúde.

**Métodos:**

estudo transversal realizado em 18 Unidades Básicas de Saúde do Nordeste brasileiro. Foram utilizados três questionários: sociodemográfico, *Maslach Burnout Inventory* e *Medical Office Survey on Patient Safety Culture*. O estudo foi aprovado por Comitê de Ética em Pesquisa.

**Resultados:**

participaram 78 trabalhadores de saúde, dos quais 64,1% apresentaram risco reduzido de *burnout*; e 11,5%, elevado risco (p=0,000). Foram identificadas como dimensões fragilizadas da cultura de segurança: Pressão no trabalho e ritmo; Apoio de gestores, administradores e líderes; Avaliação global da qualidade; e Avaliação global da segurança do paciente.

**Conclusões:**

constatou-se associação entre baixo risco de desenvolvimento da síndrome de *burnout* e avaliação positiva da cultura de segurança.

## RESUMEN

**Objetivos:**

analizar la relación entre riesgo de agotamiento profesional (*burnout*) y cultura de seguridad en la Atención Primaria de Salud.

**Métodos:**

estudio transversal realizado en 18 Unidades Básicas de Salud del Noreste brasileño. Fueron utilizadas tres encuestas: sociodemográfica, *Maslach Burnout Inventory* y *Medical Office Survey on Patient Safety Culture*. El estudio fue aprobado por Comité de Ética en Investigación.

**Resultados:**

participaron 78 trabajadores de salud, de los cuales 64,1% presentaron riesgo reducido de *burnout*; y 11,5%, elevado riesgo (p=0,000). Fueron identificadas como dimensiones de debilidades de la cultura de seguridad: Presión en el trabajo y ritmo; Apoyo de gestores, administradores y líderes; Evaluación global de la calidad; y Evaluación global de la seguridad del paciente.

**Conclusiones:**

constatada relación entre bajo riesgo de desarrollo del síndrome de *burnout* y evaluación positiva de la cultura de seguridad.

## INTRODUCTION

Actions aimed at patient safety in Primary Health Care (PHC) are still insufficient, and there is a need for a safety culture institutionalization in this care setting^(1-2)^. Furthermore, problems in communication, work relationships, and infrastructure are reported as possible predictors for PHC failures^(3-4)^.

PHC, besides being responsible for maintaining a link with its users, is the preferred gateway to the health system, providing a comprehensive and specific care to the population’s needs. In this spectrum, factors such as provision of supplies, referrals, professional ethics, technical procedures, among many others, if conducted inappropriately, may negatively impact the quality of care, leading to avoidable risks or damage^(5)^.

In this regard, it is already demonstrated that, even if in a mild form, adverse events (AEs) - incidents arising from health care that cause harm to the patient - have been identified in PHC in several countries. In Spain, for example, a retrospective analysis identified 168 AEs in adult and pediatric patients, while in England and Wales, 1,456 patient reports recorded incidents in dental care^(6-7)^.

Considering this, it is worth highlighting the importance of a safety culture in care units, that is, one in which all workers take responsibility for the safety of patients and their families - be it their colleagues or their own; and punitive culture be replaced by procedural analysis, making room for learning and improvement based on failures^(8)^.

In this sense, it is known that the damage resulting from health care is generally present in institutions where there is work overload due to lack of professionals and training^(9)^. Excessive and prolonged levels of stress at work can trigger the development of burnout syndrome. Individual characteristics associated with the work and the work environment propitiate the appearance of the multidimensional factors of the syndrome: emotional exhaustion, depersonalization, and reduced personal accomplishment^(10)^.

Occupational burnout is considered a risk, since, when exhausted during the workday, professionals tend not to provide effective care, which can cause damage to themselves, to patients, and to health services^(11-12)^.

## OBJECTIVES

To analyze the association between the risk of occupational exhaustion (burnout) and safety culture in Primary Health Care.

## METHODS

### Ethical aspects

The study was reviewed and approved by a Research Ethics Committee and followed all its required ethical precepts.

### Study design, period, and location

Cross-sectional type study with a quantitative approach, developed in accordance with the guidelines of the checklist for cross-sectional studies Strengthening the Reporting of Observational Studies in Epidemiology (STROBE)^(13)^.

It was conducted in 18 Basic Health Units (BHUs) of municipalities in the state of Ceará (CE), Brazil, between October 2019 and February 2020.

### Population and sample

The population was composed of all health workers linked to the BHUs: nurses, nursing technicians and assistants, physicians, dentists, oral health technicians, physical therapists, speech therapists, physical educators, managers, and pharmacy technicians. A non-probability convenience sampling was adopted. We included, therefore, those workers linked to the BHUs and who were present at the time of the researchers’ visit. Those who were on vacation or leave were excluded.

### Study protocol

Data collection was carried out by voluntary participation. The health workers who were present in the BHUs were invited to participate in the research, and those who accepted responded the self-administered instruments immediately or scheduled the delivery for a later date.

Initially, the research participants’ profile was evaluated through a questionnaire of sociodemographic and labor data, which included the following variables: profession and time working in the job, time working in the unit and in other places, weekly workload, and night and weekend shifts.

To assess emotional exhaustion, the translated and validated Brazilian version of the Maslach Burnout Inventory (MBI-HSS) instrument was applied^(14)^. The scale presents 22 items, nine of which are related to emotional exhaustion (EE), which assesses complaints about feeling on edge and exhausted by work; five, to depersonalization (DP), which measures impersonal responses and lack of empathy during professional activity; and eight related to personal accomplishment (PA), assessing feelings of competence and achievement of success at work. The answers were distributed on a seven-point Likert-type scale: 0) never; 1) a few times a year; 2) once a month; 3) a few times a month; 4) once a week; 5) a few times a week; and 6) every day^(14)^.

The evaluation of professional burnout was performed according to the three dimensions (EE, DP, and PA), classified as low, moderate, and high levels. In this study, we used the term Inefficacy as an indication of reduced personal accomplishment. Each subscale is assessed separately, and each has cut-off points: EE - high ≥ 27, moderate 17 to 26, low ≤ 16; DP - high ≥ 13, moderate 7 to 12, low ≤ 6; and Inefficacy - high 0 to 31, moderate 32 to 38, low ≥ 39^(15)^. However, the values proposed by the author were inversely applied in the Personal Accomplishment subscale: high ≥ 39, moderate/medium 32 to 38, and low 0 to 31. This was needed because there is still no consensus in the literature on how the syndrome classification should be performed according to the MBI-HS results; here, it is proposed that this be done according to a relationship between the subscales, as recommended by authors^(16)^.

A high index in emotional exhaustion or depersonalization, or low index in personal accomplishment, is an indication of risk of occurrence of the syndrome, generating one point in the risk score. Thus, three points indicates burnout; two points, at high risk of burnout; one point, at medium risk; and with medium/low EE or DP and medium/high PA, at low risk.

To evaluate safety culture, the translated and validated Brazilian version of the Medical Office Survey on Patient Safety Culture - (MOSPSC) questionnaire, developed in 2007 by the Agency for Health Care Research and Quality (AHRQ)^(17)^ was used. The instrument consists of 51 questions that measure 12 dimensions of the patient safety construct, upon which the interpretation of the results was based. Those with 75% positive answers were considered strong (strengthened); those with room for improvement (potential), presented positive answers lower than 75% and higher than 50%; and finally, those in which positive answers were lower than 50% were considered fragile (weakened)^(18)^.

In addition, each dimension was analyzed separately, calculating the percentage of positivity in each one. For this, the following calculation was used: [number of positive answers to the dimension items / total number of valid answers to the dimension items (positive, neutral, and negative, excluding missing data and “does not apply” or “do not know” answers)] × 100^(18)^.

### Analysis of results and statistics

Absolute and relative frequencies were used for categorical variables (sex, profession, time working in the profession and in the unit, workload in the institution and in total, professional activity in other places, night shift and work in other places). Numerical variables (age in years, mean scores of the dimensions of the MBI-HSS and MOSPSC questionnaires) were described as mean, standard deviation, median, minimum value, maximum value, and confidence interval of 95% (CI95%), when pertinent.

For hypothesis testing, considering the categorical variables, Pearson’s chi-square test and Fisher’s exact test were applied. The significance level was 5%, and the public domain software Epi Info, version 7.2.1.0 (CDC, Atlanta, USA) was used for statistical processing.

## RESULTS

A total of 78 health care workers participated in the survey, consisting of: 64 (82%) women, 42 (53.8%) single or divorced, and 36 (46.2%) married. As for the professional category, there were 21 nursing technicians (26.9%), 19 nurses (24.3%), 11 physicians (14.1%), 11 dentists (14.1%), 4 oral health assistants (5.1%), 3 nursing assistants (3.8%), 2 physical therapists (2.6%), 2 managers (2.6%), 2 pharmacy technicians (2.6%), 1 speech therapist (1.3%), 1 physical educator (1.3%), and 1 oral health technician (1.3%). The mean age was 34 years.

A large portion of the interviewees had worked in their field between one and five years (32%), the same period during which most of them worked in the Unit where they were interviewed (53.8%). As for the workload at the BHU, 63 (80.8%) worked 40 hours a week. When asked whether they worked in other places, more than half (59%) answered no; this interferes directly in the following questioning regarding total workload (considering all service locations), which is 40 hours a week for most (64.1%).

Table 1 shows the percentages obtained in each of the MBI-HSS items. Initially, analyzing the Emotional Exhaustion (EE) subscale, the items “I feel frustrated by my job” and “I feel used up at the end of the workday” draw our attention; specifically, when the last three points of the scale are added, 51.3% and 56.4%, respectively, feel this way at least a few times a month.

**Table 1:** Percentage of the relative frequency of each item of the Maslach Burnout Inventory (MBI-HSS), within the corresponding dimension, for health professionals in Primary Care (N = 78), Redenção, Ceará, Brazil, 2020

Subscale and Items	Intensity Scores - %						
	0	1	2	3	4	5	6
Emotional Exhaustion							
*I feel frustrated by my job*	23.1	19.2	6.4	24.4	2.6	15.4	8.9
*I feel used up at the end of the workday*	16.7	14.1	12.8	20.5	9.0	15.4	11.5
*I am fatigued when I get up in the morning*	37.2	12.8	10.3	17.9	6.4	6.4	9.0
*Working with people all day is a strain for me*	46.1	16.7	9.0	10.3	7.7	5.1	5.1
*I feel burned out from work*	30.8	24.4	10.3	14.1	6.4	8.9	5.1
*I feel frustrated by my job*	56.4	25.6	5.1	3.9	1.3	3.9	3.9
*I feel I am working too hard on my job*	38.5	20.5	5.1	12.8	2.6	15.4	5.1
*Working with people directly puts stress on me*	33.3	11.5	20.5	15.4	2.6	9.0	7.7
*I feel like I am at the end of my rope*	76.9	15.4	1.3	3.8	-	-	2.6
Average Percentage of the Scores - Emotional Exhaustion	39.9	16.8	9.0	13.7	4.3	8.8	6.5
Depersonalization							
*I feel I treat some patients as impersonal objects*	80.8	7.7	3.9	2.5	2.5	1.3	1.3
*I have become more callous towards people since I took this job*	62.8	7.7	9.0	5.1	3.9	5.1	6.4
*I worry that this job is hardening me emotionally*	56.4	11.5	3.9	9.0	3.8	-	15.4
*I really do not care what happens to some patients*	78.3	6.5	3.8	3.8	3.8	-	3.8
*I feel patients blame me for some of their problems*	58.9	14.1	6.4	2.6	5.1	10.3	2.6
Average Percentage of the Scores - Depersonalization	67.4	9.5	5.4	4.6	3.8	3.4	5.9
Personal Accomplishments							
*I easily understand how my patients feel about things*	-	2.6	5.1	7.7	2.6	19.2	62.8
*I deal very effectively with problems of my patients*	3.8	3.8	5.1	10.3	5.1	18.0	53.9
*I feel I am positively influencing people’s lives*	2.6	1.3	-	1.3	12.8	26.9	55.1
*I feel very energetic*	10.3	5.1	5.1	6.4	6.4	30.8	35.9
*I can easily create a relaxed atmosphere with patients*	2.6	2.6	1.3	10.3	8.9	28.2	46.1
*I feel exhilarated after working close with patients*	-	3.9	2.6	12.8	6.4	28.2	46.1
*I have accomplished many worthwhile things in this job*	3.8	-	1.3	9.0	6.4	29.5	50.0
*I deal with emotional problems very calmly*	3.9	5.1	3.9	20.5	5.1	19.2	42.3
Average Percentage of the Scores - Personal Accomplishments	3.4	3.0	3.0	9.8	6.8	25.0	49.0

*0 - Never; 1 - A few times a year; 2 - Once a month; 3 - A few times a month; 4 - Once a week; 5 - A few times a week; and 6 - Every day.*

Regarding the Depersonalization (DP) subscale, in all items, most workers (67.4%) stated never going through the situations described. However, it is worth mentioning the fact that 15.4% of the interviewees affirmed that every day they have the feeling that “I worry that this job is hardening me emotionally”. Finally, regarding the third subscale, Personal Accomplishments (PA), in this case, with results considered positive according to the higher frequency of the reported situations, the majority (74%) answered that they perceive them sometimes a week or every day. However, in the item “I deal with emotional problems very calmly”, 20.5% of the interviewees informed that it happens only a few times a month.

The MBI-HSS subscales were also analyzed according to the frequency distribution and according to the level of burnout risk. In general, most workers presented a low level of EE (59%) and DP (66.7%); and a high level of PA (56.4%). However, it is noteworthy that 20.5% of the professionals showed a high level of exhaustion, while 19.2% showed a low level of accomplishment. Regarding the risk of burnout, it is positive that 64.1% of the professionals show a reduced risk. It is, however, worth noting the presence of nine professionals with high risk. All values are shown in Table 2.

**Table 2:** Distribution of frequencies and measures of burnout level (MBI-HSS) according to the dimensions of the MBI-HSS among health professionals (N = 78), Redenção, Ceará, Brazil, 2020

Subscale		Levels			Mean Score (SD) Min - Med - Max
		High	Medium	Low	
Emotional Exhaustion	n (%)	16 (20.5)	16 (20.5)	46 (59.0)	16.0 (11.4)
	CI_95%_	[12.2 - 31.2]	[12.2 - 31.2]	[47.2 - 70.0]	0.0 - 13.5 - 46.0
*p* value			0.000		
Depersonalization	n (%)	08 (10.3)	18 (23.1)	52 (66.7)	5.1 (5.0)
	CI_95%_	[4.5 - 19.2]	[14.3 - 34.0]	[55.1 - 76.9]	0.0 - 5.0 - 18.0
*p* value			0.000		
Personal Accomplishments	n (%)	44 (56.4)	19 (24.4)	15 (19.2)	38.8 (7.1)
	CI_95%_	[44.7 - 67.6]	[15.4 - 35.4]	[11.2 - 29.7]	19.0 - 40.0 - 48.0
*p* value			0.000		
High Risk of Burnout		09 (11.5) - CI_95%_ [5.4 - 20.8]			*p* value
Moderate Risk of Burnout		19 (24.4) - CI_95%_ [15.4 - 35.4]			0.000
Reduced Risk of Burnout		50 (64.1) - CI_95%_ [52.4 - 74.7]			

*n - absolute value; (%) relative value; CI95% - 95% confidence interval; SD - standard deviation; Min. - minimum score; Med. - median score; Max. - maximum score; Statistics: Pearson's chi-square test.*

Moving on to the MOSPSC, when analyzing the averages of the patient safety culture dimensions, the following were identified as weakened: Work pressure and pace (28.2%); Owner, managing partners, leadership support (39.2%); Overall ratings on quality (47.6%); and Overall rating on patient safety (44.9%). Other dimensions’ classifications are described in Table 3.

**Table 3:** Dimensions of patient safety culture among Primary Health Care professionals (N = 78) according to the average percentage of positivity of the responses, Redenção, Ceará, Brazil, 2020

Safety Culture Dimensions	Mean^*^	SD	95%CI	Dimensions Classification
*Section A:* Patient safety and quality issues	69.1	12.3	(61.4-76.7)	Potentiality
*Section B:* Information exchange with other settings	60.0	7.5	(53.4-66.6)	Potentiality
*Section C:* Working in your medical office				
Teamwork	77.3	2.7	(74.7-79.9)	Strengthened
Work pressure and pace	28.2	12.0	(16.4-39.9)	Weakened
Staff training	55.5	3.9	(51.1-59.9)	Potentiality
Procedures established in the unit	51.5	15.9	(35.9-67.0)	Potentiality
*Section D:* Communication and follow-up				
Open communication	63.2	14.1	(49.4-77.0)	Potentiality
Communication on errors	59.1	22.1	(37.4-80.8)	Potentiality
Patient follow-up	86.6	8.0	(78.7-94.4)	Strengthened
*Section E:* Owner, Managing Partners, Leadership Support	39.2	7.9	(31.4-47.0)	Weakened
*Section F:* Your medical office				
Organizational learning	76.5	8.6	(66.7-86.3)	Strengthened
Overall perception of patient safety and quality	76.6	2.3	(74.3-78.9)	Strengthened
*Section G:* Overall ratings				
Overall ratings on quality	47.6	11.3	(37.7-57.6)	Weakened
Overall rating on patient safety	44.9	-	(33.6-56.6)	Weakened

* *Data presented as mean percentage of positive answers per dimension of the MOSPSC instrument; SD - standard deviation; CI95% - 95% confidence interval.*

Considering the above, it was pertinent to analyze the relationship between the risk of burnout syndrome and patient safety culture. To do so, the variables were dichotomized to apply Fisher’s test to validate the analysis. Table 4 shows the relationship with the Overall ratings on quality, and most participants considered the dimension as a “potentiality” or “weakened” (67.9%). However, it is noteworthy that, among those who considered it positive, most (24) present low or moderate risk for the syndrome.

**Table 4:** Relationship between the classification of risk of burnout and the Overall ratings on quality - Section G1 expressed by health professionals (N = 78), Redenção, Ceará, Brazil, 2020

Risk Classification	Overall ratings on quality		*p* value
	Strengthened	Potentiality/Weakened	
Elevated	01 (11.1)	08 (88.9)	0.152
Reduced/Moderate	24 (34.8)	45 (65.2)	
Total	25 (32.1)	53 (67.9)	

* Values expressed as absolute and relative - n (%);

** Pearson’s chi-square test - one cell (25.0%) with expected count less than 5; Fisher’s Exact test (p = 0.258).

In Table 5, similar results can be seen, related to the Overall rating on patient safety. Here, the majority considered the dimension negative/neutral (55.1%). Likewise, 34 of the 35 who considered it positive were classified with low or moderate risk of developing burnout.

**Table 5:** Relationship between the classification of risk of burnout and the Overall rating on patient safety - Section G2 expressed by health professionals (N = 78), Redenção, Ceará, Brazil, 2020

Risk Classification	Overall rating on patient safety		*p* value
	Positive	Negative/Neutral	
Elevated	01 (11.1)	08 (88.9)	0.030
Reduced/Moderate	34 (49.3)	35 (50.7)	
Total	35 (44.9)	43 (55.1)	

* Values expressed as absolute and relative - n (%);

** Pearson’s chi-square test - two cells (50.0%) with expected count less than 5; Fisher’s Exact test (p = 0.037).

## DISCUSSION

After analyzing the relationship of the data between the two questionnaires, in general, the quality of care was evaluated as weakened, that is, more effective actions are needed for change; or as having potential, meaning it’s got potential to become strengthened. However, it is noticeable the greater relation of the high risk of burnout with the participants’ negative evaluation of this dimension. There is still no strong evidence in the literature between the quality of care and the syndrome^(19)^, but, although with moderate effects, the attrition of the healthcare professional would affect the patient’s satisfaction^(20)^, demonstrating the novelty of the research and the importance of the discussion on the theme.

Patient safety obtained similar evaluations among individuals with low or moderate burnout risk. However, it was observed that most participants with high risk assessed it as negative or neutral. Here we highlight the relationship between stress and incidents involving safety, as well as its influence on professional dissatisfaction and desire to quit the job^(21)^.

Other issues such as work overload, problems with management or coworkers, and with the work structure, can also contribute to professional dissatisfaction, in addition to disappointment with the work performed and exhaustion after the workday^(22)^. Some consequences may result from this, such as the lack of professional accomplishment and, in turn, a decrease in the quality of care provided^(23)^.

The reported aspects can culminate in the hardening of professionals, as identified in the present study. The factor can be considered worrisome, as the harsher treatment towards people can lead to the breakdown or even the non-formation of bonds, violating the principles of work in PHC, like longitudinality, which deals with the monitoring of users over time. Herein lies the paradox, as it is inherent to health workers the involvement, care, and interaction with patients^(24)^.

At the same time, the low levels of emotional exhaustion and depersonalization, associated with high professional accomplishment, characterize the reduced level of burnout in the present sample. This finding diverges from the results of studies with PHC professionals in different countries, since, in these, increased levels of professional exhaustion and depersonalization and, consequently, burnout syndrome were evidenced.

In Chile, for example, individuals with high emotional exhaustion (29.7%) and depersonalization (28.9%) and low levels of accomplishment (25.5%) were identified^(22)^. In Oman, attention is drawn to the depersonalization scale, in which 38.2% had a high level^(25)^. Moreover, in China, in a study with 951 primary care providers and 48 physicians in primary health care institutions, the results were more expressive for emotional exhaustion and depersonalization, with 33.12% and 41.43%, respectively^(26)^. In the United States, research with 1,273 health professionals from 154 primary care facilities identified burnout in 31.6% of physicians, 18.9% of clinical support staff, and 17.5% of administrative staff^(27)^.

In a research, providers who experienced higher levels of anxiety and withdrawal were three times more likely to report burnout compared to those who experienced low levels in these domains. Thus, understanding individual behaviors and attitudes toward change can help leaders and policymakers develop strategies to reduce burnout among health care professionals^(27)^.

While investigating the factors contributing to burnout and low professional accomplishment among primary care professionals in Massachusetts (USA), participants described their workloads as excessive, with increasing “office work”, reflecting unreasonable expectations. They felt demoralized by their working conditions, devalued by local institutions and the healthcare system, and conflicted in their daily work lives. When sharing their perspectives on factors contributing to burnout, the interviewees described dissonance between their professional values and the realities of Primary Care, an incompatibility between authority and responsibility, and a feeling of undervaluation^(28)^.

While assessing the prevalence of emotional exhaustion, depersonalization, and low accomplishment in a sample of 1,110 primary care nurses, researchers found a high prevalence of emotional exhaustion (28%) (95%CI = 22%-34%), high depersonalization (15%) (95%CI = 9%-23%), and low personal accomplishment (31%) (95%CI = 6%-66%). They concluded that problems such as emotional exhaustion and low personal accomplishment are very common among Primary Care nurses, diverging from the findings of the present study; while depersonalization is less prevalent^(29)^.

Analyzing the dimensions of patient safety culture among Primary Health Care professionals, most were classified with potential for improvement. However, there is an emphasis on those classified as strengthened: Teamwork, Organizational learning, Overall perception of patient safety and quality, and Patient follow-up.

The first two aspects were also considered positive in a study carried out in the Brazilian Midwest. With a sample of 246 health professionals, the dimensions Teamwork (73.1%) and Organizational learning (62.9%) were positively evaluated^(30)^. In contrast, in Kuwait, the responses of 6,602 employees of Primary Care Centers indicated the need for improvement in the dimension Overall perception of patient safety and quality^(31)^.

The results presented may be influenced by other aspects related to safety culture. Regarding the weakening in the Work pressure and pace dimension, the result may be due to several factors, such as high workload, an element identified in most professionals interviewed. At the same time, the high patient demand and, often, the small number of professionals that make up the health teams can generate pressure to perform the activities^(32)^.

Meanwhile, it is worrisome that the dimension Owner, managing partners, leadership support is weakened. For adequate safety in care, everyone’s involvement is necessary, and this starts with a well-structured leadership that encourages actions based on learning, which involves, for continuous improvement in this aspect, monitoring and listening to the professionals working directly in the assistance^(33-34)^.

Another point is the fact that the Overall ratings on quality and Overall rating on patient safety dimensions are weakened. Thus, strengthening patient safety and quality of care is important, since, when there are failures, harm can be caused to patients. One of the ways of strengthening these dimensions is through professional qualification of those involved, with training on the subject through strategies such as permanent or continuing education^(35)^. Managers should also focus on strengthening the work environment to improve the organizational capacity for change and address the high levels of anxiety and burnout experienced by PHC professionals^(27)^.

It is also worth noting that the dimensions Open communication and Communication on errors have the potential to become strengthened. Communication is one of the most relevant aspects of patient safety within health institutions. Failures in the communication process can represent a barrier, because they prevent the checking of information or the clarification of doubts. At the same time, if performed effectively, it becomes a facilitator, because teamwork, based on the exchange of experiences and collaboration, contributes positively to higher quality care^(36)^.

Considered as an important health problem that can affect several professional categories, burnout requires individual interventions and institutional support to prevent its evolution and/or to solve it. It is worth reflecting, therefore, if the positive results observed are related to the investment in PHC in the state where the research was carried out, especially in small municipalities.

Initiatives such as the *Programa Nacional de Melhoria do Acesso e da Qualidade da Atenção Básica* (PMAQ-AB) [National Program for Improvement of Access and Quality of Primary Care], implemented in Ceará between 2012 and 2014, generated more significant results in municipalities with 10,000 to 20,000 inhabitants, among which include those investigated in this study^(37)^. Thus, it can be inferred that the availability of resources contributed to a faster improvement in the infrastructure of the BHUs, while enabling, in the medium term, transformations in the work process and organizational culture, thus impacting the quality of services provided^(38)^.

### Study limitations

This study was limited by a smaller than expected sample size, resulting from the difficulty of handing out and getting the questionnaires back from the health professionals. Moreover, there were reports of fatigue when answering them, due to their length and the complexity of some items, which may have influenced the resolution of the questions.

### Contributions to the field of Health

We highlight the innovation of this research as a strong point, since there are no reports of studies of this type in PHC. Therefore, the results may become subsidies for advances in the approach of the theme with professionals at this level of care. It is noteworthy that, in Family Health teams, nurses are leaders and can approach and focus their actions on the precepts of patient safety in PHC.

## CONCLUSIONS

An association was found between low risk of developing burnout syndrome and positive evaluation of the safety culture in health care units. Considering that patient safety is one of the dimensions of quality, the relationships demonstrated indicate the need for effective interventions to prevent the effects of the syndrome from harming patient care. Since PHC is the main gateway to the Health System and to the bond between users and health professionals, the promotion of quality care that does not cause harm to the subjects involved should be prioritized.

## Data Availability

https://doi.org/10.48331/scielodata.OQO4SG
